# Automatic assessment of body composition in children with lymphoma: results of a [^18^F]FDG-PET/MR study

**DOI:** 10.1007/s00330-024-10957-4

**Published:** 2024-07-16

**Authors:** Chiara Giraudo, Celeste Cavallin, Marta Pillon, Elisa Carraro, Giulia Fichera, Diego Cecchin, Pietro Zucchetta

**Affiliations:** 1 Unit of Advanced Clinical and Translational Imaging, Department of Cardiac, Thoracic, Vascular Sciences and Public Health—DCTV, University of Padova, Padova, Italy; 2 Hospital of Cittadella, Cittadella, Italy; 3 Pediatric Hematology, Oncology and Stem Cell Transplant Division, Department of Woman’s and Child’s Health, University of Padua, Padua, Italy; 4 Pediatric Radiology Unit, Azienda Ospedale-Università Padova, Padova, Italy; 5 Nuclear Medicine Unit, Department of Medicine—DIMED, University of Padova, Padova, Italy

**Keywords:** Magnetic resonance imaging, Muscle, Adipose tissue, Children, lymphoma

## Abstract

**Objectives:**

To use Dixon-MR images extracted from [^18^F]FDG-PET/MR scans to perform an automatic, volumetric segmentation and quantification of body composition in pediatric patients with lymphoma.

**Materials and methods:**

Pediatric patients with lymphoma examined by [^18^F]FDG-PET/MR at diagnosis and restaging were included. At each time point, axial fat and water Dixon T1w images of the thighs were automatically segmented and muscle volume, subcutaneous, intramuscular, and intermuscular fat volume were quantified. The metabolic activity of the largest nodal lesion and of muscles and subcutaneous fat was recorded. The paired samples *t*-test and Spearman’s correlation coefficient were applied to evaluate potential differences between the two time points and the relationship between metabolic and body composition metrics, respectively. By logistic regression analysis, the prognostic role of the investigated variables was assessed. The applied significance level was *p* < 0.05 for all analyses.

**Results:**

Thirty-seven patients (mean age ± SD 14 ± 3-years-old; 20 females) matched the inclusion criteria. After chemotherapy (interval between the two PET/MR scans, 56–80 days; median 65 days), muscle volume significantly decreased (629 ± 259 cm^3^ vs 567 ± 243 cm^3^, *p* < 0.001) while subcutaneous, intramuscular and intermuscular fat increased (476 ± 255 cm^3^ vs 607 ± 254 cm^3^, *p* < 0.001; 63 ± 20 cm^3^ vs 76 ± 26 cm^3^, *p* < 0.001; 58 ± 19 cm^3^ vs 71 ± 23 cm^3^, *p* < 0.001); the metabolic activity of the main nodal lesion, muscles, and subcutaneous fat significantly decreased (*p* < 0.05, each). None of the examined variables acted as predictors of the response to treatment (*p* = 0.283). A strong correlation between BMI and subcutaneous fat volume at diagnosis (*r* = 0.675, *p* < 0.001) and restaging (*r* = 0.600, *p* < 0.001) emerged.

**Conclusions:**

The proposed method demonstrated that pediatric patients with lymphoma undergo muscle loss and an increase of subcutaneous fat during treatment.

**Clinical relevance statement:**

The proposed automatic and volumetric MR-based assessment of body composition in children with lymphoma can be used to monitor the effect of chemotherapy and may guide tailored exercise programs during chemotherapy.

**Key Points:**

- *T1w Dixon images can be used for the automatic segmentation and quantification of body composition*.
- *Muscle and subcutaneous fat volume do not act as predictors of the response to treatment in children with lymphoma*.
- *Chemotherapy induces changes in body composition in children with lymphoma*.

## Introduction

It has been widely demonstrated that the assessment of body composition plays a significant role in oncological patients. In fact, numerous studies have shown how low muscle mass or sarcopenia may affect the prognosis in various types of tumors or overweight and obesity might be a risk factor for cancer contributing to metabolic and endocrine changes [1–5]. Moreover, poor body composition metrics can be associated with increased treatment-related toxicity as shown in patients with early breast cancer receiving anthracyclines-taxane [6]. Controversial results were obtained regarding the impact of chemotherapy on body composition since, for instance, Jang et al demonstrated an increase in fat and a decrease in muscle mass in Korean women with breast cancer [7] while in similar populations van Der Berg depicted only modest changes, not substantially different from patients without cancer [8], and Jung and colleagues highlighted the impact of menopause rather than chemotherapy [9].

The impact of body composition on tumor response, as well as the effect of chemotherapy on fat and muscle mass, has also been investigated in oncological children. For instance, Kellerman et al found nutritional depletion and skeletal muscle loss in children undergoing chemotherapy, as well as an increase in weight, body mass index (BMI), and fat mass, especially in those with hematological malignancies [10]. Moreover, Wadhwa et al identified an association between low muscle density at computed tomography (CT) and hematological toxicities in children with lymphoma or rhabdomyosarcoma [11].

In terms of the type of assessment, in addition to the anthropometric evaluation relying for instance on weight, height, BMI, abdominal circumference, and skinfold measurement, in the last decades, the application of various radiological techniques such as dual-energy X-ray absorptiometry, ultrasound, CT, and magnetic resonance (MR) provided robust results allowing a precise tissue characterization and distinction between muscle and fat mass [12, 13]. In particular, MR imaging via the application of Dixon sequences, which are based on the acquisition of two or more echoes at different echo times (TE), enables the evaluation and quantification of body composition metrics [12, 14]. For example, it has been successfully used to quantify fat in obese subjects and to assess the effect of exercise on muscle composition [15, 16]. Even hybrid imaging demonstrated to contribute to the muscle assessment of oncological patients, in fact, as suggested by Roshdy et al, high muscle metabolic activity expressed as 18F-fluorodeoxyglucose ([^18^F]FDG) uptake may act as a predictor of response to therapy in head and neck cancer, reflecting the overall inflammatory status associated with tumors [17].

Despite the growing interest in body composition for adults and children with tumors, up to now, to the best of our knowledge, a study automatically quantifying body composition in pediatric patients with lymphoma under treatment was still missing.

Thus, the aim of this project was to evaluate body composition using automatic, volumetric segmentation and quantification of muscle and fat mass via Dixon-MR images extracted by the [^18^F]FDG positron emission tomography/MR (PET/MR) scan of children and adolescents with lymphoma and evaluate muscle and subcutaneous changes during chemotherapy.

## Materials and methods

### Study design and PET/MR protocol

Children and adolescents (i.e., < 21-years-old) with nodular sclerosing Hodgkin lymphoma referring to our tertiary center who underwent a [^18^F]FDG-PET/MR for staging and restaging after the first cycle of treatment were included in this retrospective, review board-approved study. All patients were examined by a fully integrated 3-T PET/MR scanner (Biograph mMR; Siemens, Erlangen, Germany) with high-performance gradient systems (45 mT/m) and equipped with a phased-array body coil. All patients underwent the standardized pediatric protocol applied in our unit including whole-body axial and coronal high-resolution Controlled Aliasing in Parallel Imaging Results in Higher Acceleration (CAIPIRINHA) Dixon T1weighted images (repetition time (TR) 3.85 ms, TE 1.23 ms, and 3 mm slice thickness), axial T2- Half Fourier Single-shot Turbo spin-Echo (HASTE; TR 1600 ms, TE 95 ms, and 5 mm slice thickness with 6 mm spacing between slices), axial Turbo Inversion Recovery Magnitude (TIRM; TR 7790 ms, TE 76 ms, inversion time 220 ms, and 4 mm slice thickness), and Diffusion-Weighted Imaging (DWI; two *b* values, 50 and 800, TR 4900 ms, TE 53 ms, five averages, 5 mm slice thickness with 6 mm space between slices). Regarding the PET, the images (5 min/bed position) were acquired 60 min after intravenous injection of [^18^F]FDG (≥ 6 h fasting, 3 MBq/kg), following the European Association of Nuclear Medicine recommendations, from the head to the toes [18, 19]. The total scanning time for each patient was of around 45 min, depending on patient height.

### Image analysis

**Fig. 1:**
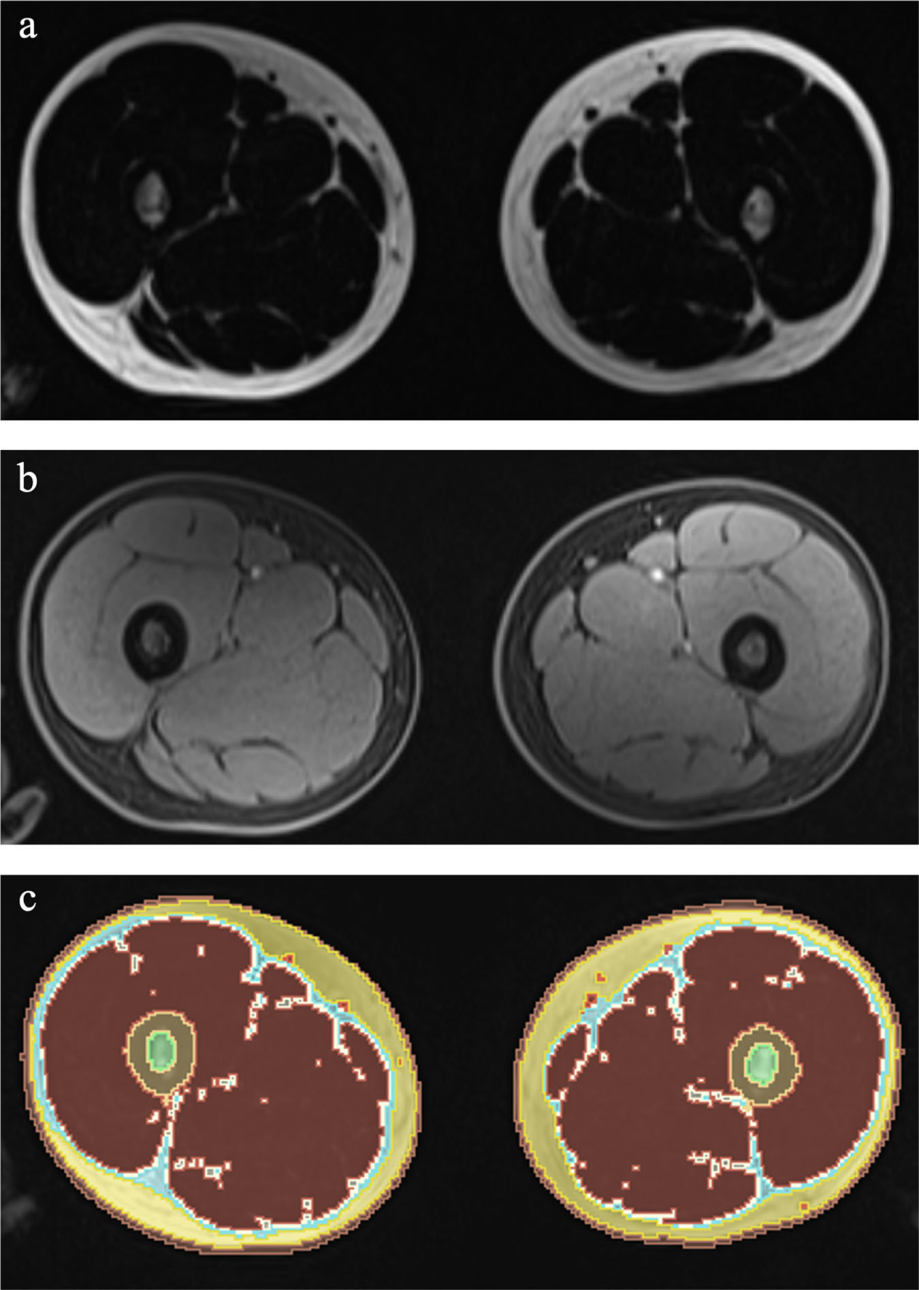
Axial fat only (**a**) and water only (**b**), and the tissue segmentations (**c**) performed by 3D slicer of a 15-year-old boy with Hodgkin lymphoma examined at staging by PET/MR

**Fig. 2:**
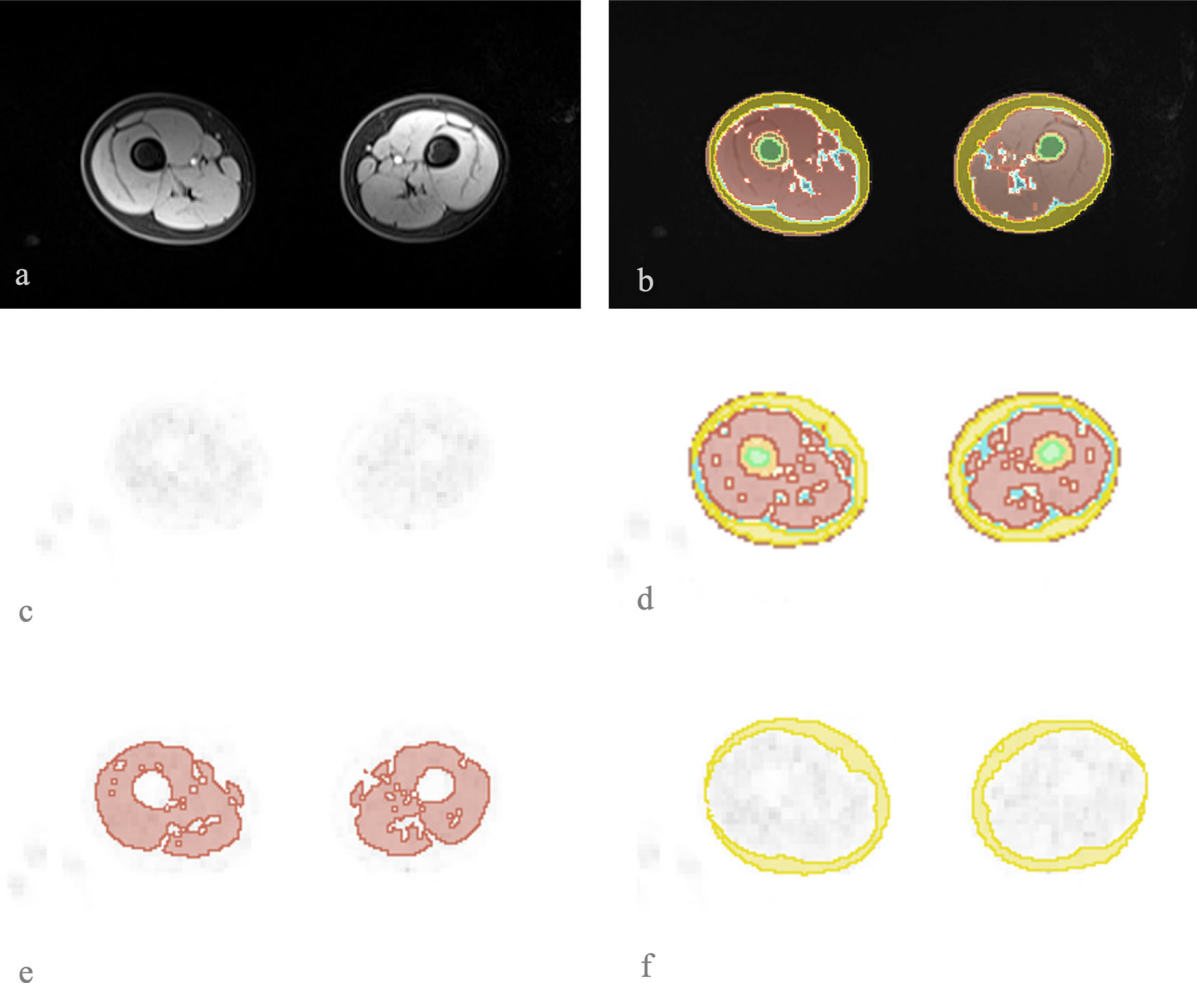
In **a**, example of the axial water only, used together with the fat only, to perform the tissue segmentation represented in **b**, which was then applied to the PET map, in **c**. In **d**, the overall tissue segmentation on the PET dataset, in **e** the segmentation of muscle only, and in **f** the segmentation of the subcutaneous fat, both were then used to extract the SUV values

One radiologist with four years of experience in oncological and musculoskeletal imaging, extracted between 10 and 30 slices (overall cranio-caudal extension between 3 cm and 9 cm), according to patient’s age and height, from the axial CAPIRINHA Dixon T1w images of the thigh of each enrolled patient at both time points. The fat and water only datasets were processed with the plugin “TisSeg” of the open-source software 3D Slicer (www.slicer.org) (Fig. 1). By this plugin an automatic segmentation at diagnosis and at restaging of the thighs of each patient was achieved and volumes of muscles, subcutaneous, intramuscular, and intermuscular fat extracted. The same segmentation has been applied to the PET dataset to extract SUVmax and SUVmean of muscles and subcutaneous fat tissue at both time points (Fig. 2). Moreover, a manual region of interest has been drawn on the largest nodal lesion and its metabolic activity was collected (i.e., SUVmax and SUVmean).

Demographics and clinical information including treatment, weight, height, and BMI were also collected for each enrolled patient at diagnosis and restaging.

### Statistical analyses

Descriptive statistics were performed. The paired samples t-test was applied to evaluate if a difference in muscle and/or subcutaneous fat volume and/or standardized uptake value (SUV) values occurred between the two time points (i.e., diagnosis and restaging).

Logistic regression analysis was applied to assess if any of the investigated variables, including demographics, acted as predictors of the response to therapy.

The Spearman’s correlation coefficient was used to investigate the relationship between the metabolic activity (i.e., SUVmax and SUVmean) of the largest nodal lesion and muscle and subcutaneous fat, as well as the relationship of body composition metrics (i.e., muscle volume, subcutaneous, intramuscular, and intermuscular fat) and BMI at staging and restaging.

Segmentations and quantitative MR metrics extraction were repeated by the same rater after eight weeks and the intra-rater reliability was assessed by the intraclass correlation coefficient (ICC) considering values > 0.750 as excellent [20].

All analyses were performed by SPSS (v.28, IBM Armonk, NY, USA) applying *p* < 0.05 as the significance level.

## Results

**Fig. 3:**
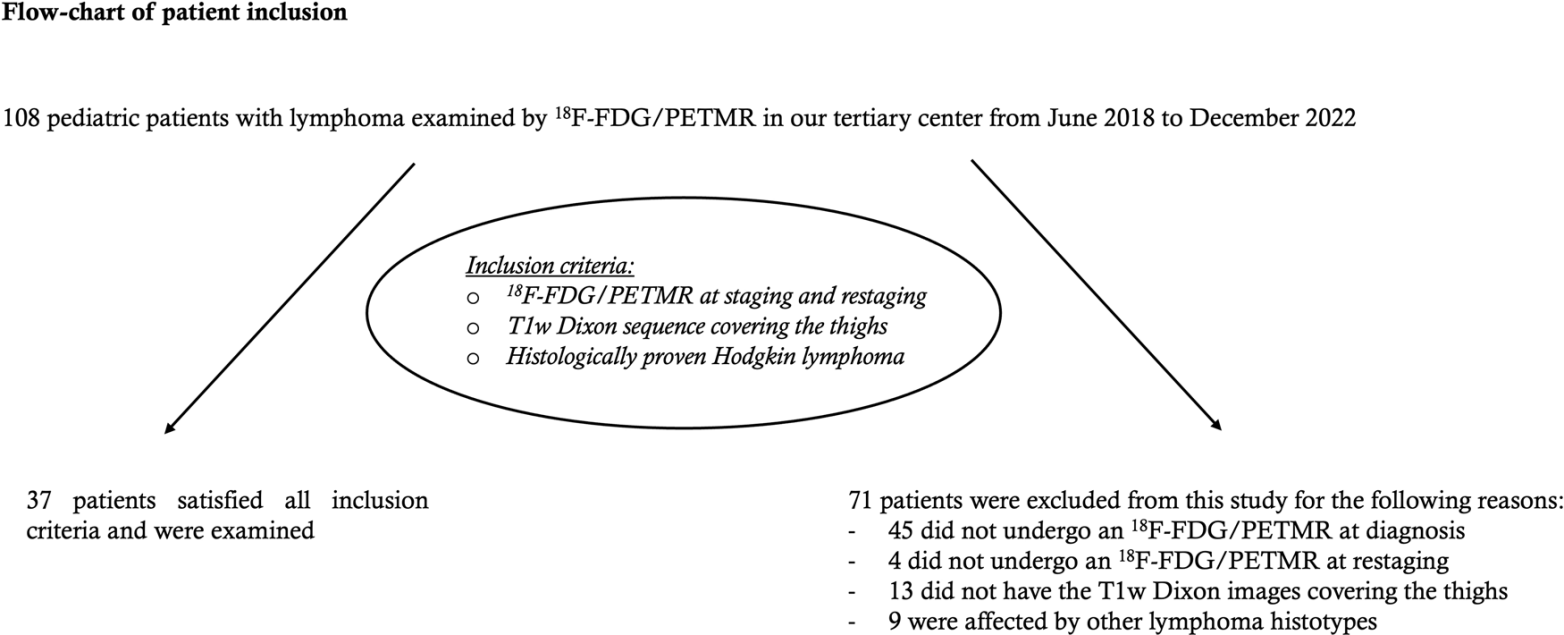
Flowchart of the applied inclusion criteria

Overall, 37 patients (mean age 14 ± 3-years-old; 20 females) matched the inclusion criteria and were examined. Figure 3 shows the flowchart of patient inclusion, and the characteristics of the examined population are summarized in Table 1.

**Table 1:** Characteristics of the examined population

	Value
Age at diagnosis (range, years-old)	5–18
Gender (female:male)	20:17
Height at diagnosis (mean ± SD, cm)	162 ± 15
Weight (mean ± SD, Kg)	53 ± 16
BMI at diagnosis (range, Kg/m^2^)	15–33
Average days between first and second PET/MR scan	67.4
Treatment	EuroNet-PHL-C2 38
Number of responders Number of not responders	28 9

Twenty-eight were responders after the first cycle of treatment according to the EuroNet-PHL-C2 38 protocol. Referring to the tables of the World Health Organization for BMI in children, all children except two were in the 50th percentile; indeed, one boy was in the underweight range (i.e., 15-years-old and 15 BMI) and one girl was in the overweight range (i.e., 17-years-old and 33 of BMI) [21].

After chemotherapy (interval between the two PET/MR scans, 56–80 days; median 65 days), muscle volume significantly decreased (629 ± 259 cm^3^ vs 567 ± 243 cm^3^, *p* < 0.001). On the contrary, the subcutaneous fat increased during the same interval (476 ± 255 cm^3^ vs 607 ± 254 cm^3^, *p* < 0.001).

**Fig. 4:**
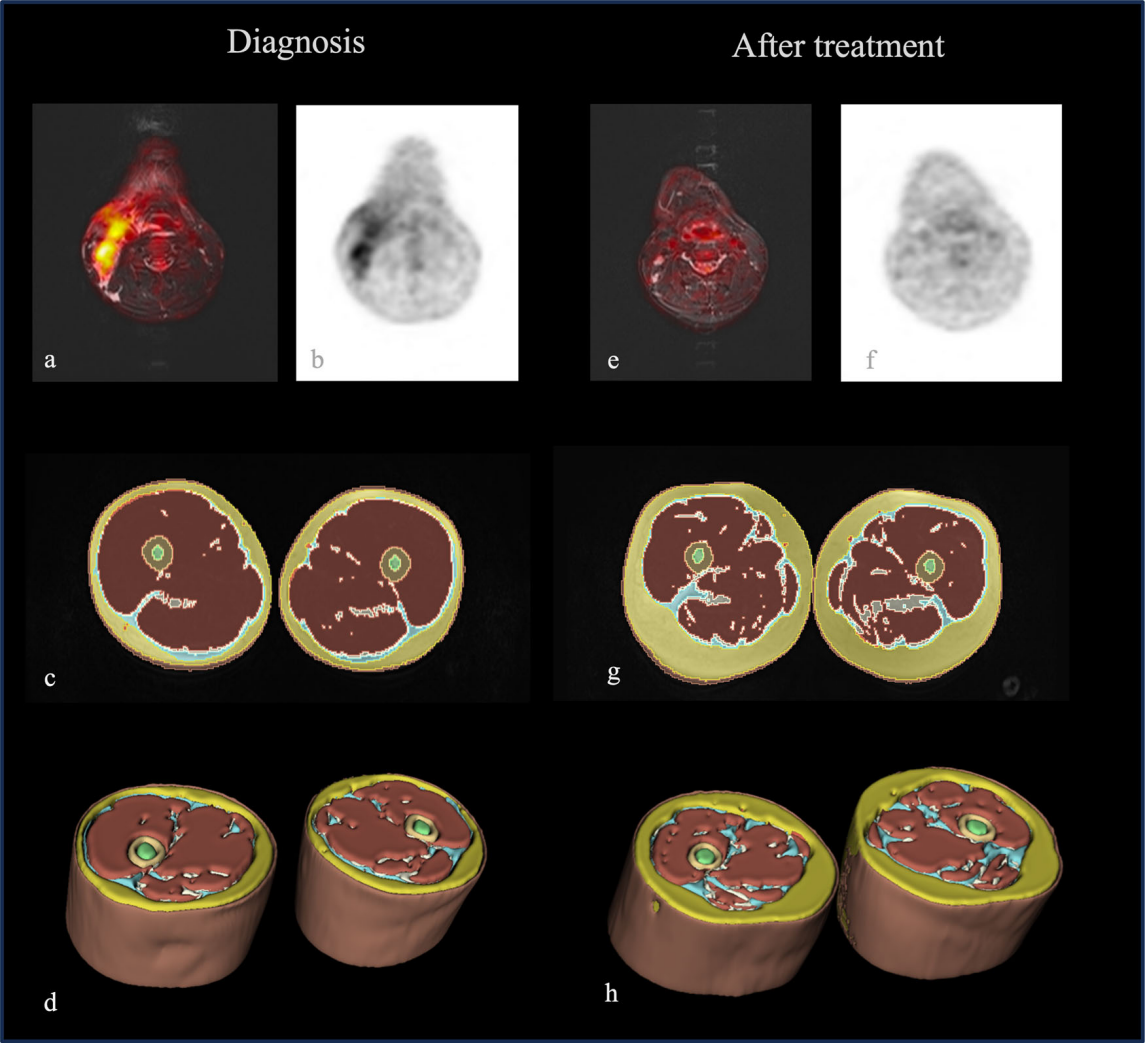
Sixteen-year-old boy with Hodgkin lymphoma examined by [^18^F]FDG-PETMR for staging and restaging. In **a** and **b**, the fused axial STIR and the PET map show hypermetabolic cervical lymph nodes. In **c** and **d**, the 2D and 3D segmentations of his thighs at staging. After treatment, a full response was achieved as demonstrated by the reduced size and uptake of the cervical lymph nodes (**e** and **f**), and in the thighs, a significant increase of the subcutaneous fat, as well as a loss of muscle mass occurred (**g** and **h**, respectively)

Similarly, intramuscular and intermuscular fat volume significantly increased after treatment (63 ± 20 cm^3^ vs 76 ± 26 cm^3^, *p* < 0.001 and 58 ± 19 cm^3^ vs 71 ± 23 cm^3^, *p* < 0.001, respectively) (Fig. 4).

**Fig. 5:**
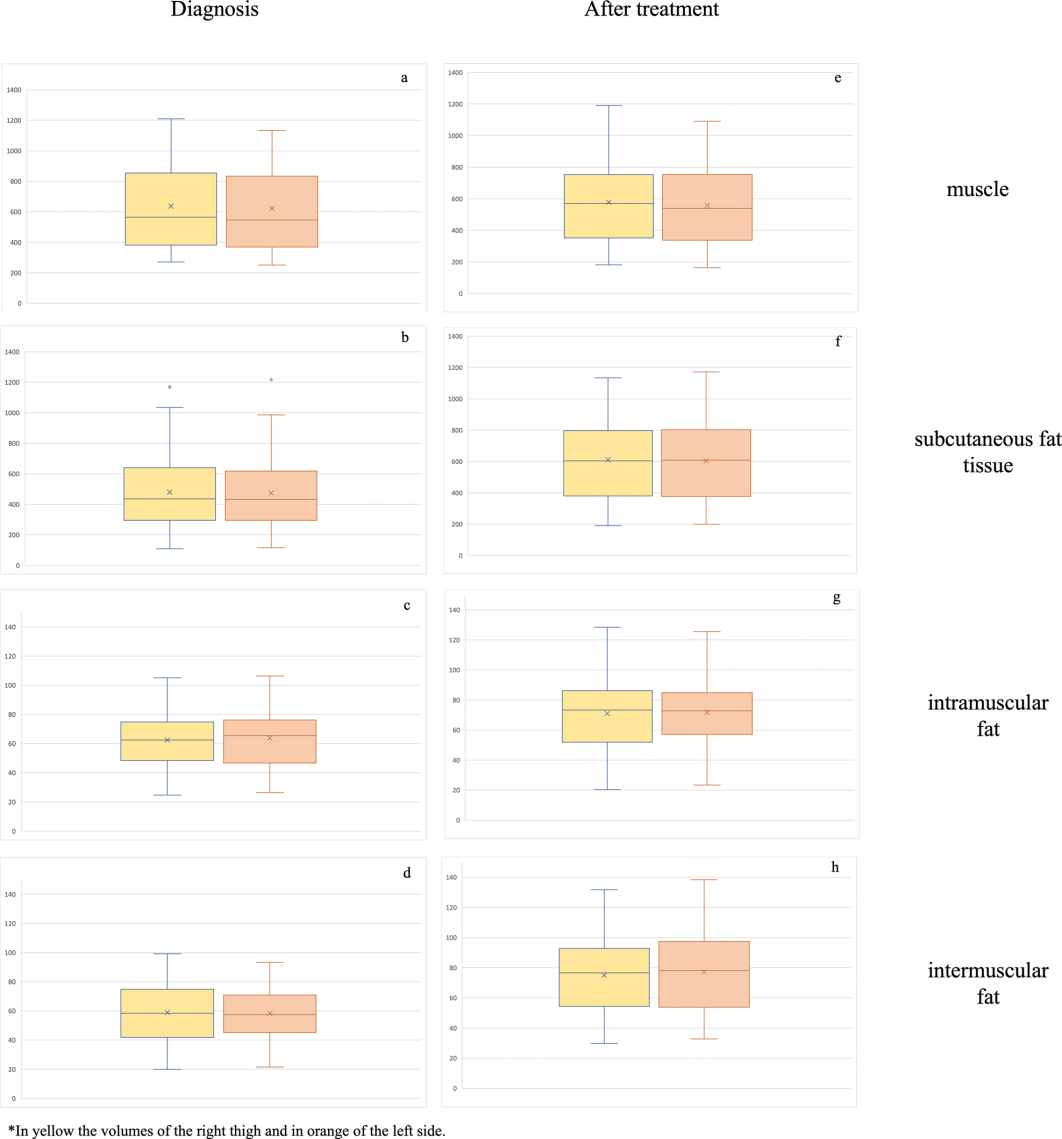
Box plots showing the differences of body composition metrics between the right and left thigh at diagnosis and after chemotherapy (**a**–**h**). The right side showed greater muscle volume at diagnosis and restaging (**a** and **e**) and the intramuscular fat was greater on the left side at restaging (**g**) (*p* < 0.05, each)

Considering each thigh, the right side showed greater muscle volume at diagnosis (637.5 ± 266 cm^3^ vs 622 ± 254 cm^3^ *p* = 0.011) and restaging (578 ± 250 cm^3^ vs 557 ± 237 cm^3^, *p* < 0.001). The intramuscular fat was greater on the left side at staging, although not significantly (62 ± 20 cm^3^ vs 64 ± 21 cm^3^, *p* = 0.052), but this trend became significant at restaging (right 75 ± 25 cm^3^ vs left 77 ± 26 cm^3^, *p* = 0.002). No other differences occurred at both time points (*p* > 0.05, each) (Fig. 5).

SUVmax and SUVmean of muscles and subcutaneous fat significantly decreased after treatment (SUVmax 2.7 ± 0.4 vs 1.5 ± 0.3, *p* < 0.001 and SUVmean 0.5 ± 02 vs 0.3 ± 0.1, *p* < 0.001).

**Fig. 6:**
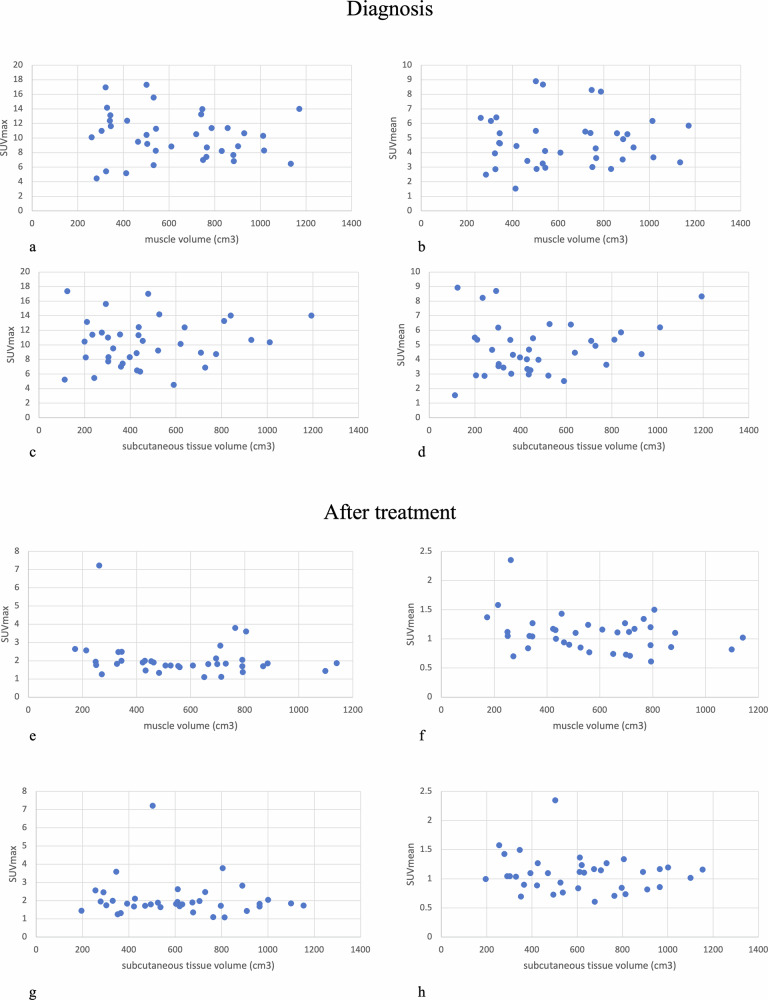
Scatter plots showing the lack of statistically significant correlation between SUVmax and SUVmean and body composition metrics (**a**–**h**)

On average, a statistically significant decrease of SUVmax and SUVmean of the main nodal lesion between staging and restaging was observed (SUVmax 10 ± 3 vs 2 ± 1 and SUVmean 5 ± 2 vs 1 ± 0.3, respectively, *p* < 0.001 each). The Spearman coefficient did not show any significant correlation between SUVmax and/or SUVmean and muscle and subcutaneous fat volume computed by MR, at diagnosis and restaging (*p* > 0.05, each; Fig. 6).

Weight and BMI increased between the two time points (53 ± 16 Kg vs 55 ± 16 Kg, *p* = 0.014 and 19.8 ± 4 vs 20 ± 4 Kg/m^2^, *p* = 0.012). Height did not change between the two PET/MR scans (on average 162 ± 15 cm, *p* = 1.00).

A strong correlation emerged between BMI and subcutaneous fat volume at diagnosis (*r* = 0.675, *p* < 0.001) and restaging (*r* = 0.600, *p* < 0.001) while a moderate correlation emerged between BMI and intramuscular fat volume at diagnosis (*r* = 0.489, *p* = 0.002) and restaging (*r* = 0.401, *p* = 0.014); a weak correlation occurred between BMI and intermuscular fat volume at diagnosis (*r* = 0.360, *p* = 0.029) and restaging (*r* = 0.396, *p* = 0.015). No significant correlations occurred between BMI and muscles at diagnosis (*r* = 0.212, *p* = 0.207) and restaging (*r* = 0.269, *p* = 0.107).

The logistic regression analysis demonstrated that none of the variables included in the model (i.e., age, gender, height, weight, BMI, and muscle volume, as well as subcutaneous, intramuscular and intermuscular fat volume, SUVmax, and SUVmean of the largest nodal lesion and of muscles and subcutaneous fat) acted as a predictor of the response to treatment (*p* = 0.283).

The volumetric tissue quantitation showed perfect repeatability for all extracted variables (ICC = 1.00, each).

## Discussion

This is the first study assessing volumetric body composition by MR images extracted from PET/MR scans in children and young adolescents with lymphoma using an automatic open-source method. Our results, showing a decrease in muscle volume and an increase in subcutaneous, intramuscular, and intermuscular fat, are in line with the previous literature based on clinical assessment and/or radiological evaluations by US and CT [7, 10, 22]. Indeed, as previously mentioned, Kellerman et al recently showed that in the first six months of chemotherapy, an increase in BMI, weight, and fat mass as well as a loss of skeletal muscle mass occur especially in children with hematological malignancies [10].

To get further insights in the occurring changes, the comparison among sides, showed greater muscle volume on the right side and a predominance, especially after treatment, of intramuscular fat on the left side, which may just reflect the effect of the dominant side [23]. In addition, the significant correlation between muscle fat infiltration and subcutaneous fat volume with BMI is in agreement with previous results applying various imaging techniques in different areas of the body [24–26].

Although we did not perform any histological correlation, we can assume that the effect of chemotherapy on body composition, as already described in the literature, might be due to pro-atrophy mechanism, abnormal mitochondrial metabolism, and reduced protein anabolism which induce muscle loss [22]. In this direction, it would be also interesting to further assess by MR the association between low skeletal muscle density and chemotherapy-related toxicity identified by Wadhwa et al using CT in children with lymphoma or rhabdomyosarcoma [11].

Although a prognostic role of body composition has been demonstrated for several types of cancer and specific groups of patients, such as post-menopausal women with breast cancer in which obesity has been associated with a higher risk of death [5, 27], the lack of this type of evidence in our study is not surprising. Indeed, the BMI at diagnosis was in the range of the 95th percentile for all patients except two and we are dealing with a pediatric population that is not affected by the typical muscle loss due to aging [28]. Despite this, applying a synergistic approach including also radiomics could reveal different prognostic results. In fact, as nicely demonstrated in the recent literature, novel quantitative biomarkers combining clinical and radiological information may lead to combined biomarkers with a better prognostic value [29]. While this approach exceeded the aim of our study, it should be certainly considered for future research.

Independently from patients’ age and from the impact on overall survival, as suggested by Pin et al, preserving muscle mass, and applying pro-anabolic strategies should be further promoted, in oncology [22].

Last, regarding the SUV, taking into account all difficulties related to the attenuation map in PET/MR, our results are in the range of previous PET/CT and PET/MR studies [30–32] and the significant decrease after treatment suggests a reduction of the inflammation which may accompany cancer, as already reported in the literature [17].

Several limitations may affect this study. Unfortunately, we could not evaluate the relationship between our MR quantitative data and laboratory tests (e.g., creatine phosphokinase) or muscle index tests (e.g., Barthel index) because they are not routinely collected in this group of patients. Further studies including these parameters and, in the case also bioptic samples, may provide new insights regarding the structural muscle changes.

We have not included additional time points and the end-of-treatment scans because not all patients underwent further controls. This drawback is associated with the retrospective design of the study. Future prospective projects are expected to overcome this limitation. Considering that in the pediatric population, small changes in the amount of physical activity have a positive impact on body composition, we may expect after the end of chemotherapy a regression of the changes we identified, but a focused project is needed [33].

We have not performed a comparison with healthy children of the same age to assess the potential impact of physiological changes due to growth, but it would have not been possible to scan by PET/MR and expose to unjustified radiation exposure healthy individuals. Moreover, previous studies like the one of Murphy et al comparing healthy children and children with cancer showed that children with cancer have low body mass and an increased fat mass [34].

In conclusion, the hereby proposed automatic method of assessment of body composition in children with lymphoma under treatment demonstrated to be robust and reliable. Moreover, it confirmed that this group of patients suffers from muscle loss and an increase in subcutaneous fat during treatment. Further studies including the end of treatment evaluation and the potential role of tailored exercise programs are highly encouraged.

## Abbreviations

BMI: Body mass index
CT: Computed tomography
[^18^F]FDG: 18F-fluorodexoxyglucose
MR: Magnetic resonance
PET: Positron emission tomography
SUV: Standardized uptake value
TE: Echo time
TR: Repetition time

## References

[1] McSweeney DM, Raby S, Radhakrishna G et al (2023) Low muscle mass measured at T12 is a prognostic biomarker in unresectable oesophageal cancers receiving chemoradiotherapy. Radiother Oncol 186:109764 37385375 10.1016/j.radonc.2023.109764

[2] Hatt J, Smart TFF, Hardy EJ et al (2023) The impact of low muscle mass on prognosis following neoadjuvant chemotherapy for resectable locally advanced rectal cancer: a systematic review and meta-analysis. JCSM Clin Rep 8:27–35

[3] Rier HN, Jager A, Sleijfer S, Maier AB, Levin MD (2016) The prevalence and prognostic value of low muscle mass in cancer patients: a review of the literature. Oncologist 21:1396–1409 27412391 10.1634/theoncologist.2016-0066 PMC5189631

[4] Yip C, Dinkel C, Mahajan A, Siddique M, Cook GJ, Goh V (2015) Imaging body composition in cancer patients: visceral obesity, sarcopenia and sarcopenic obesity may impact on clinical outcome. Insights Imaging 6:489–497 26070723 10.1007/s13244-015-0414-0 PMC4519815

[5] Caan BJ, Cespedes Feliciano EM, Kroenke CH (2018) The importance of body composition in explaining the overweight paradox in cancer-counterpoint. Cancer Res 78:1906–1912 29654153 10.1158/0008-5472.CAN-17-3287 PMC5901895

[6] Shachar SS, Deal AM, Weinberg M et al (2017) Body composition as a predictor of toxicity in patients receiving anthracycline and taxane-based chemotherapy for early-stage breast cancer. Clin Cancer Res 23:3537–3543 28143874 10.1158/1078-0432.CCR-16-2266 PMC5511549

[7] Jang MK, Park S, Park C, Doorenbos AZ, Go J, Kim S (2022) Body composition change during neoadjuvant chemotherapy for breast cancer. Front Oncol 12:941496 36091109 10.3389/fonc.2022.941496 PMC9458921

[8] van den Berg MMGA, Kok DE, Visser M et al (2020) Changes in body composition during and after adjuvant or neo-adjuvant chemotherapy in women with breast cancer stage I–IIIB compared with changes over a similar timeframe in women without cancer. Support Care Cancer 28:1685–1693 31290019 10.1007/s00520-019-04951-6 PMC7036066

[9] Jung GH, Kim JH, Chung MS (2020) Changes in weight, body composition, and physical activity among patients with breast cancer under adjuvant chemotherapy. Eur J Oncol Nurs 44:101680 31756674 10.1016/j.ejon.2019.101680

[10] Kellerman I, Blaauw R, Schoeman J, Kruger M (2023) Changes in anthropometrical status and body composition in children with cancer during initial chemotherapy. Pediatr Hematol Oncol 40:659–672 37092844 10.1080/08880018.2023.2201299

[11] Wadhwa A, Adams KM, Dai C et al (2022) Association between body composition and chemotherapy-related toxicity in children with lymphoma and rhabdomyosarcoma. Cancer 128:1302–1311 34847257 10.1002/cncr.34043

[12] Giraudo C, Cavaliere A, Lupi A, Guglielmi G, Quaia E (2020) Established paths and new avenues: a review of the main radiological techniques for investigating sarcopenia. Quant Imaging Med Surg 10:1602–1613 32742955 10.21037/qims.2019.12.15 PMC7378089

[13] Simoni P, Guglielmi R, Aparisi Gómez MP (2020) Imaging of body composition in children. Quant Imaging Med Surg 10:1661–1671 32742959 10.21037/qims.2020.04.06 PMC7378095

[14] Feuerriegel GC, Marcus RP, Sommer S, Wieser K, Bouaicha S, Sutter R (2023) Fat fractions of the rotator cuff muscles acquired with 2-point Dixon MRI: predicting outcome after arthroscopic rotator cuff repair. Invest Radiol. 10.1097/RLI.0000000000001024 10.1097/RLI.0000000000001024 PMC11882186 37707864

[15] Berglund J, Johansson L, Ahlström H, Kullberg J (2010) Three-point Dixon method enables whole-body water and fat imaging of obese subjects. Magn Reson Med 63:1659–1668 20512869 10.1002/mrm.22385

[16] Grimm A, Nickel MD, Chaudry O et al (2019) Feasibility of Dixon magnetic resonance imaging to quantify effects of physical training on muscle composition—a pilot study in young and healthy men. Eur J Radiol 114:160–166 31005168 10.1016/j.ejrad.2019.03.019

[17] Roshdy E, Ahmed T, Abdelhafez Y (2022) Pretherapy muscle [^18^F]FDG uptake: association with outcome in advanced head and neck cancer. J Nucl Med 63:3107

[18] Veit-Haibach P, Ahlström H, Boellaard R et al (2023) International EANM-SNMMI-ISMRM consensus recommendation for PET/MRI in oncology. Eur J Nucl Med Mol Imaging 50:3513–3537 37624384 10.1007/s00259-023-06406-x PMC10547645

[19] Boellaard R, Delgado-Bolton R, Oyen WJ et al (2015) FDG PET/CT: EANM procedure guidelines for tumour imaging: version 2.0. Eur J Nucl Med Mol Imaging 42:328–354 25452219 10.1007/s00259-014-2961-x PMC4315529

[20] Cicchetti DV (1994) Guidelines, criteria, and rules of thumb for evaluating normed and standardized assessment instruments in psychology. Psychol Assess 6:284–290

[21] BMI for age (5-19). World Health Organization - WHO. Available via https://www.who.int/tools/growth-reference-data-for-5to19-years/indicators/bmi-for-age. Accessed 14 Dec 2023

[22] Pin F, Couch ME, Bonetto A (2018) Preservation of muscle mass as a strategy to reduce the toxic effects of cancer chemotherapy on body composition. Curr Opin Support Palliat Care 12:420–426 30124526 10.1097/SPC.0000000000000382 PMC6221433

[23] Kulas AS, Schmitz RJ, Shultz SJ et al (2018) Bilateral quadriceps and hamstrings muscle volume asymmetries in healthy individuals. J Orthop Res 36:963–970 28755488 10.1002/jor.23664

[24] Hernandez R, Younan Y, Mulligan M et al (2019) Correlation between subcutaneous fat measurements in knee MRI and BMI: relationship to obesity and related co-morbidities. Acta Radiol Open 8:2058460119853541 31218080 10.1177/2058460119853541 PMC6560802

[25] Nadeem B, Bacha R, Gilani SA (2018) Correlation of subcutaneous fat measured on ultrasound with body mass index. J Med Ultrasound 26:205–209 30662152 10.4103/JMU.JMU_34_18 PMC6314101

[26] Mehdorn M, Schnarkowski B, Moulla Y et al (2023) Visceral obesity determined in routine preoperative CT scans predicts risk of postoperative burst abdomen. Sci Rep 13:21429 38052856 10.1038/s41598-023-48714-0 PMC10697964

[27] Chan DSM, Vieira AR, Aune D et al (2014) Body mass index and survival in women with breast cancer-systematic literature review and meta-analysis of 82 follow-up studies. Ann Oncol 25:1901–1914 24769692 10.1093/annonc/mdu042 PMC4176449

[28] Larsson L, Degens H, Li M et al (2019) Sarcopenia: aging-related loss of muscle mass and function. Physiol Rev 99:427–511 30427277 10.1152/physrev.00061.2017 PMC6442923

[29] Delgado Bolton RC, Calapaquí Terán AK, Fanti S, Giammarile F (2023) The concept of strength through synergy applied to the search of powerful prognostic biomarkers in gastroesophageal cancer: an example based on combining clinicopathological parameters, imaging-derived sarcopenia measurements, and radiomic features. Clin Nucl Med 48:156–157 35961366 10.1097/RLU.0000000000004357

[30] Lyons K, Seghers V, Sorensen JI et al (2015) Comparison of standardized uptake values in normal structures between PET/CT and PET/MRI in a tertiary pediatric hospital: a prospective study. AJR Am J Roentgenol 205:1094–1101 26496558 10.2214/AJR.15.14304

[31] Kershah S, Partovi S, Traughber BJ et al (2013) Comparison of standardized uptake values in normal structures between PET/CT and PET/MRI in an oncology patient population. Mol Imaging Biol 15:776–785 23632951 10.1007/s11307-013-0629-8 PMC4822407

[32] Karunanithi S, Soundararajan R, Sharma P, Naswa N, Bal C, Kumar R (2015) Spectrum of physiologic and pathologic skeletal muscle (18)F-FDG uptake on PET/CT. AJR Am J Roentgenol 205:W141–W149 26001118 10.2214/AJR.14.13457

[33] Carrel AL, Clark RR, Peterson SE, Nemeth BA, Sullivan J, Allen DB (2005) Improvement of fitness, body composition, and insulin sensitivity in overweight children in a school-based exercise program: a randomized, controlled study. Arch Pediatr Adolesc Med 159:963–968 16203942 10.1001/archpedi.159.10.963

[34] Murphy AJ, White M, Davies PS (2010) Body composition of children with cancer. Am J Clin Nutr 92:55–60 20484453 10.3945/ajcn.2010.29201

